# ProteinHistorian: Tools for the Comparative Analysis of Eukaryote Protein Origin

**DOI:** 10.1371/journal.pcbi.1002567

**Published:** 2012-06-28

**Authors:** John A. Capra, Alexander G. Williams, Katherine S. Pollard

**Affiliations:** 1 J. David Gladstone Institutes, University of California, San Francisco, California, United States of America; 2 Institute for Human Genetics and Division of Biostatistics, University of California, San Francisco, California, United States of America; University of California, San Diego, United States of America

## Abstract

The evolutionary history of a protein reflects the functional history of its ancestors. Recent phylogenetic studies identified distinct evolutionary signatures that characterize proteins involved in cancer, Mendelian disease, and different ontogenic stages. Despite the potential to yield insight into the cellular functions and interactions of proteins, such comparative phylogenetic analyses are rarely performed, because they require custom algorithms. We developed *ProteinHistorian* to make tools for performing analyses of protein origins widely available. Given a list of proteins of interest, *ProteinHistorian* estimates the phylogenetic age of each protein, quantifies enrichment for proteins of specific ages, and compares variation in protein age with other protein attributes. *ProteinHistorian* allows flexibility in the definition of protein age by including several algorithms for estimating ages from different databases of evolutionary relationships. We illustrate the use of *ProteinHistorian* with three example analyses. First, we demonstrate that proteins with high expression in human, compared to chimpanzee and rhesus macaque, are significantly younger than those with human-specific low expression. Next, we show that human proteins with annotated regulatory functions are significantly younger than proteins with catalytic functions. Finally, we compare protein length and age in many eukaryotic species and, as expected from previous studies, find a positive, though often weak, correlation between protein age and length. *ProteinHistorian* is available through a web server with an intuitive interface and as a set of command line tools; this allows biologists and bioinformaticians alike to integrate these approaches into their analysis pipelines. *ProteinHistorian*'s modular, extensible design facilitates the integration of new datasets and algorithms. The *ProteinHistorian* web server, source code, and pre-computed ages for 32 eukaryotic genomes are freely available under the GNU public license at http://lighthouse.ucsf.edu/ProteinHistorian/.

“This is a *PLoS Computational Biology* Software Article.”

## Introduction

The proteins present in a species arose at a range of evolutionary times, and the context of a protein's origin can provide information about its cellular functions and interactions [1], [2]. Young proteins in a range of species differ from their older counterparts in many functionally relevant traits. Young yeast proteins have fewer interaction partners and are enriched for different functions than older proteins [2], [3]. Young proteins in many clades experience weaker and more variable selective pressures than older proteins [4]–[8] and have less complex regulatory programs [9]. These findings suggest that knowledge of a protein's evolutionary origins is informative when studying its cellular roles, adaptability, and regulation.

By assigning a phylogenetic “age” to each protein in a species based on the distribution of evolutionarily related sequences across other species, several recent studies identified enrichment for proteins of specific ages in biologically relevant conditions. For example, the proteins expressed during developmental stages that exhibit morphological similarity across phyla were found to be older than those expressed during stages that exhibit species-specific morphologies [10]. Analyses of proteins associated with diseases also found striking similarities between phylogenetic patterns and previously observed phenotypic patterns [11], [12]. Proteins associated with cancer exhibit enrichment for two origins: during the emergence of multicellularity and at the last common ancestor of all cellular life. The functional disruptions caused by mutations to proteins in these two categories were found to reflect their ages [11]. An early prototype of the *ProteinHistorian* tool was used in a recent investigation of the evolutionary origins of the sirtuins, a protein family that contains several histone deacetylases. By computing ages for all seven human sirtuins and several of their substrates, we successfully predicted a novel substrate for a mitochondrial sirtuin [13]. This example suggests that protein age patterns could be used more generally to predict protein-protein interaction pairs.

In this article, we present *ProteinHistorian* —an integrated web server, database, and set of command line tools for carrying out eukaryotic protein age analyses in a simple, intuitive pipeline. Our approach is similar to that commonly used for Gene Ontology annotation enrichment analysis [14]—given an input protein set of interest, its phylogenetic distribution is compared to that of a relevant background set. Since different definitions of protein “age” may be appropriate in different contexts, *ProteinHistorian* offers several strategies for estimating ages from phylogenetic patterns that make use of different ancestral family reconstruction algorithms [15], [16] and pre-existing databases of evolutionary relationships [17]–[19]. These options allow advanced users to infer ages that are best suited to their application. *ProteinHistorian* currently estimates ages for eukaryotic proteins only, because of the potential confounding effects of horizontal gene transfer on inferring evolutionary trees in prokaryotes. In addition, it does not produce explicit ancestral genome reconstructions.

To illustrate the use of *ProteinHistorian*, we describe the computation of ages for all proteins in 32 eukaryotic species using two ancestral family reconstruction algorithms and several different evolutionary databases. We then contrast the age distributions of several protein sets of interest. In the first analysis, we demonstrate that proteins with high expression in human—compared to chimpanzee and rhesus macaque expression patterns—are significantly younger than those with human-specific low expression. Next, we show how external annotation databases can be used to test hypotheses about the origins of protein functions. Taking functional annotations from the Gene Ontology [20], [21], we find that proteins with regulatory functions are significantly younger than those with catalytic functions. Finally, to demonstrate how additional quantitative attributes of proteins can be integrated into *ProteinHistorian* analysis, we compare protein age and length in 24 metazoa and fungi. These tests confirm previous results and reveal a modest, though consistent and significant, positive correlation between protein age and length in nearly all species.

## Design and Implementation

### Capabilities

**Figure 1:**
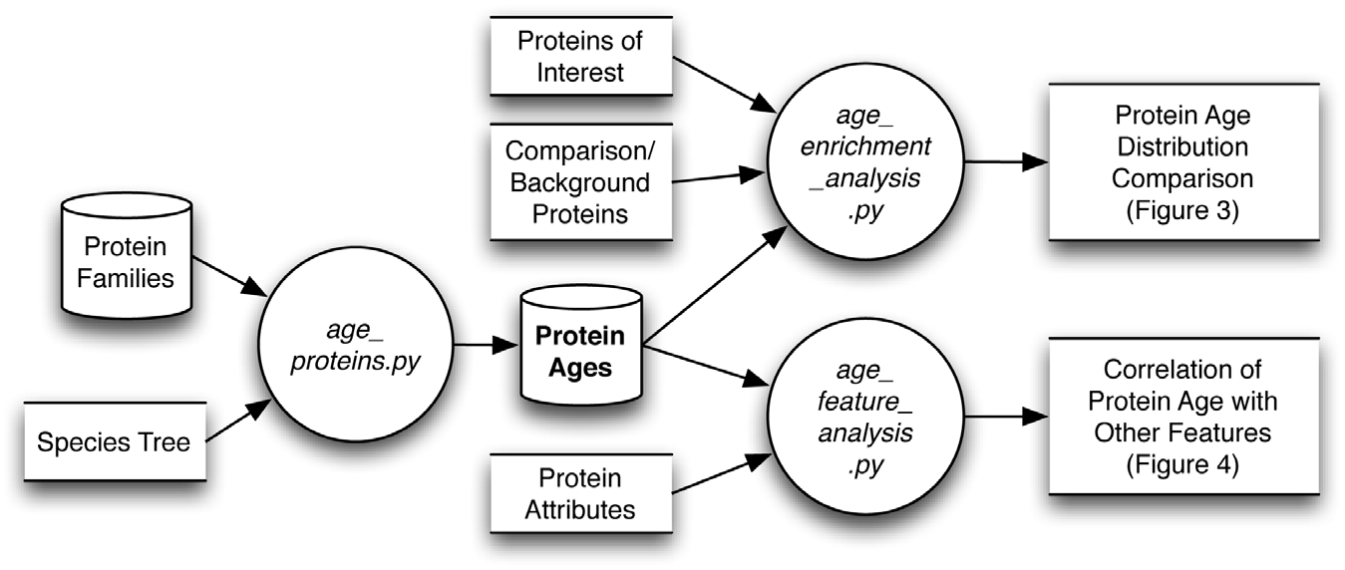
Data flow diagram representing the inputs and outputs for the *ProteinHistorian* analysis pipeline. Three python programs (circles) perform the *ProteinHistorian* analyses. age_proteins.py analyzes cross-species protein family databases, such as those provided by PPOD or our domain database, and a corresponding species tree to estimate ages for all the proteins (bold). The resulting age databases are available for download and serve as the basis for all other analyses. Protein age distribution comparisons and enrichment tests, as in Figure 3, are performed by age_enrichment_analysis.py. Correlations between protein ages and other features, as in Figure 4, are computed by age_feature_analysis.py. The *ProteinHistorian* web server provides a user-friendly interface to many of the analyses performed by these programs.

The *ProteinHistorian* web server and command line tools can perform a variety of protein age enrichment analyses (Figure 1). In the simplest case, a user inputs a set of proteins of interest in a eukaryotic species. *ProteinHistorian* first computes the phylogenetic ages of the proteins. The user can choose among several different pre-computed age databases; see the next section for more discussion of the computation and interpretation of protein age in different contexts.

Next, the distribution of ages in the input set is compared to the background of all proteins in the species. Statistically significant differences between the overall distribution of ages, as well as differences within each specific age group are identified. *ProteinHistorian* also computes phylogenetic profiles [1]—patterns of presence and absence of homologous proteins across species—for all proteins.

This basic analysis can be extended in several ways. First, the sets of proteins can be controlled to allow different types of comparisons. Rather than comparing a single protein set to the entire proteome, the user can input two protein sets to directly compare their age distributions. One list can be a proper subset of the other, which serves as a more specific background than the set of all proteins, or the two lists can be disjoint subsets of the proteome. We use the latter approach in our example comparison of proteins with high and low human-specific expression. Second, the ages of a set of proteins can be compared directly to other quantitative protein characteristics, providing further insight into potential relationships between protein function and origin. If the user inputs quantitative measurements for a set of proteins (e.g., length, essentiality, evolutionary rate), *ProteinHistorian* computes the correlation of this feature with age.

**Figure 2:**
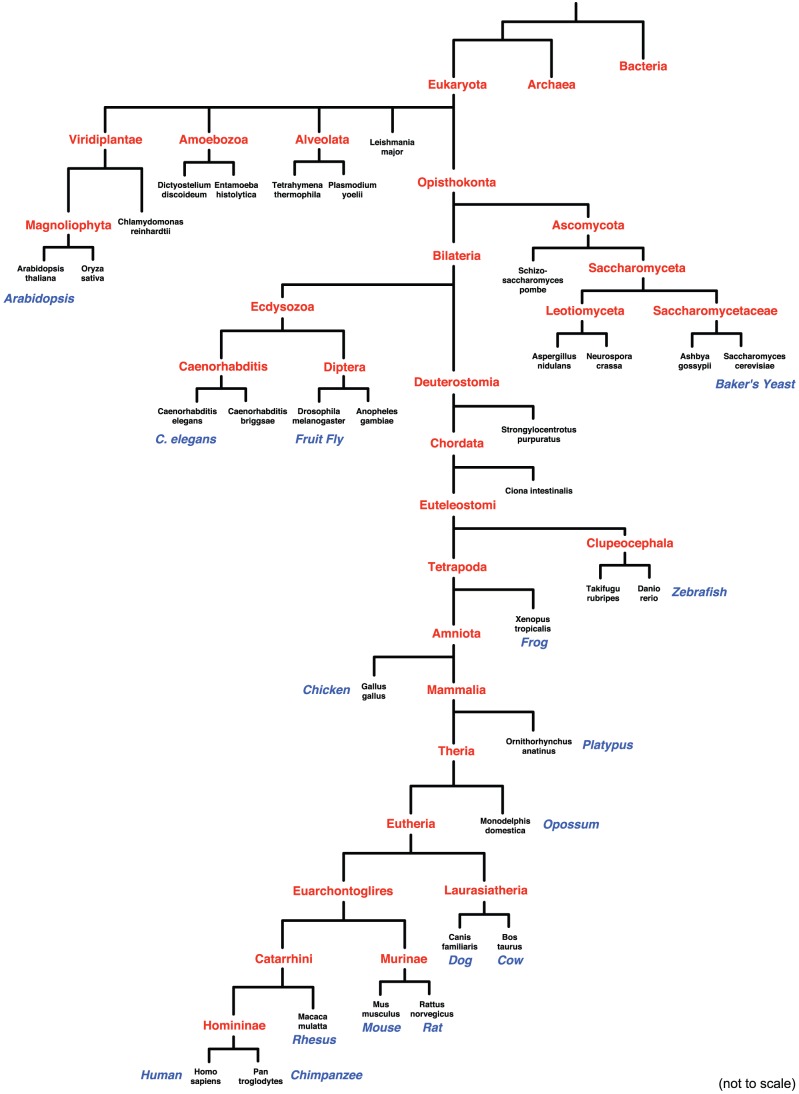
Species tree used with the PPOD PANTHER database analysis. Ages were computed for each protein in each leaf species in this species tree. The internal (red) nodes give the taxon names used as potential protein ages in this analysis. Several bacteria and archaea were considered outgroups in the analysis, but are not shown in this figure. The polytomy at the Eukaryota reflects current uncertainty about the early history of the Eukaryotes [41]. This species tree is based on the NCBI Taxonomy database [25], but has been adapted to reflect recent research on the Ecdysozoa clade [26], [27]. Branch length estimates (in millions of years ago) were taken from the TimeTree database [28]; however, for ease of visualization, the tree is not drawn to scale.

We provide for download estimated ages for all proteins from the 32 eukaryotic species listed in Figure 2, including many model organisms. However, since we anticipate that advanced users will want to compute ages for proteins and species that may not have been integrated into currently available databases of evolutionary relationships, the command line version of *ProteinHistorian* provides resources for extending the pre-computed databases. Users can build new protein age databases from their own species trees and protein families; they can also select subsets of species from existing data sets for analysis. The flexibility provided by our open source command line programs, which are described in more detail in the Implementation section, enables advanced users to develop their own analysis pipelines.

To our knowledge, *ProteinHistorian* is the only tool available for protein age enrichment analysis and visualization. However, there are other web servers that perform complementary phylogenetic analyses based on existing protein family databases. For example, the PhyloPat [22] server performs regular expression searches on phylogenetic profiles. Similarly, PhyloPro [23] allows a user to visualize the evolutionary trajectory of a set of proteins, such as a metabolic pathway, across many eukaryotes. Other tools, such as GLOOME [24], infer the evolutionary history of a protein family using a range of phylogenetic models.

### The estimation and interpretation of protein age

All analyses performed by *ProteinHistorian* rely on the assignment of a phylogenetic age to each protein in a species. Given the diverse contexts in which phylogenetic analysis can be applied, there is not a single consistent definition of protein age or origin. In this section, we describe the different ways *ProteinHistorian* computes ages to help users select the best parameters for their analyses and interpret their results. We provide several pre-computed protein age sets for each species on the *ProteinHistorian* web server in the hope that users will rarely have to compute their own ages.

The protein ages computed by *ProteinHistorian* are based on three inputs: a species tree, a protein family database, and an ancestral family reconstruction algorithm. Ages are defined with respect to the species tree; each protein is assigned to the branch in the tree on which its family is estimated to have appeared. This calculation is based on the ancestral history produced by running a family reconstruction algorithm on the protein family. Thus, the notion of “evolutionary relatedness” encapsulated in the family database has a major effect on the meaning of the resulting age. We now describe each of the inputs to the age estimation pipeline.

#### Species trees

The species tree used in all analyses presented here (Figure 2) is based on the NCBI taxonomy database [25], but when necessary, it has been modified to reflect recent research, e.g., the Ecdysozoa clade [26], [27]. Divergence time estimates in millions of years ago (mya) for each internal node in the species tree are consensus estimates from the literature taken from the TimeTree database [28]. TimeTree's expert estimates are used when available; otherwise the TimeTree weighted average of divergence time estimates in the literature are used. It is important to note that a protein could have appeared at any time along the branch to which it is assigned, so the divergence time estimate reported is a lower bound. In addition, though the topology of this species tree is relatively non-controversial and the branch lengths reflect current research, users should keep in mind that the resulting age estimates are sensitive to the tree used. The command line version of *ProteinHistorian* allows users to input their own trees.

#### Protein family databases

Each protein family database provides a partition of all proteins in all species represented in the tree into evolutionarily related families. The particular database selected defines the meaning of “relatedness” between two proteins in the resulting set of ages. *ProteinHistorian* uses several sets of protein family predictions from the Princeton Protein Orthology Database (PPOD) [18]. PPOD provides family predictions for all proteins in the 12 genomes of the GO Reference Genome Project [29] made with MultiParanoid [30], OrthoMCL [31], a Naive Ensemble (Nens) clustering-based consensus of the MultiParanoid and OrthoMCL predictions, and PPOD's own Jaccard clustering-based approach. The input to these methods is an all-versus-all BLAST sequence similarity matrix. MultiParanoid, OrthoMCL, and Nens aim to create families of orthologous proteins, while the Jaccard clustering produces larger families of more distantly related protein sequences. OrthoMCL predictions are also available for the 48 genomes (including 32 eukaryotes) in version 7 of the PANTHER database [17]. Unless otherwise noted, this family database is used in the example analyses presented here.

Several recent analyses assigned ages to proteins based on the phylogenetic distribution of the functional subdomains that they contain [3], [10]. To enable domain-based analysis in *ProteinHistorian*, we analyzed the phylogenetic distribution of all Pfam domains [19] across all species in the PANTHER database. We then used the estimated domain ages to create two different age databases: one in which each protein is assigned the age of its youngest Pfam domain and one in which each protein is given the age of its oldest domain. Proteins with no predicted domains are considered specific to the species in which they occur. We also make the estimated ages for protein domains available, so that users can perform age analyses on individual domains rather than entire proteins.

*ProteinHistorian* also includes several species-specific sets of protein ages that have been used in previous studies. For baker's yeast, *Saccharomyces cerevisiae*, age estimates used in a recent study of novel genes are available [2], as well as ages based on predicted fungal orthogroups [32]. For human, phylostratigraphic estimates of protein age [33] are included. As they become available, we will incorporate additional age databases that are likely to be of wide utility to *ProteinHistorian* users.

#### Ancestral history reconstruction algorithms

Two reconstruction algorithms, Dollo parismony and Asymmetric Wagner parsimony are currently available to infer the series of gains and losses that best explains the observed phylogenetic distribution of proteins in a family. Dollo parsimony is based on the assumption that gaining a complex structure is much more rare than losing one. Thus, it assumes that there was a single gain event for each family, potentially followed by many losses in specific lineages. In other words, under Dollo parsimony, a family's origin is the most recent common ancestor (MRCA) of all species in which it is observed. In contrast, asymmetric Wagner parsimony allows multiple gain and loss events as well as the ability to set weights on the relative likelihood of these events. By default, *ProteinHistorian* uses a relative gain penalty of 1. Since we focus on eukaryotic species in which horizontal gene transfer is rare, this largely serves to prevent false positives in the protein family databases from biasing age distributions.

Given these inputs, each family's evolutionary history is reconstructed. Then, each protein is assigned as its age the branch in the species tree on the path between its species (the leaf) and the root in which its family first appeared according to the reconstruction.

In general, the protein ages calculated by *ProteinHistorian* can be interpreted as estimates of the time at which the ancestors of proteins with recognizable homology first evolved; however, the exact nature of that similarity will vary from database to database. For example, relationships in the PPOD databases are based on varying levels of detectable sequence similarity over the entire protein, while the Pfam databases indicate the existence of similarity over individual functional domains. Sequence similarity across proteins from different species suggests a similar common function, but it does not necessarily imply that the ancestral proteins' functions were the same as those observed in current species.

Since the family databases and reconstruction algorithms provided in *ProteinHistorian* are based on different assumptions, analyzing the same protein set based on different family partitions may yield different results. For example, the use of Wagner parsimony produces a younger protein distribution than Dollo parsimony, since it allows multiple gain events to explain families with patchy existence profiles (Supplementary Figure S1). Similarly, using the Jaccard clustering families will on average produce an older age distribution than either OrthoMCL or InParanoid (Supplementary Figure S2), since it aims to detect more distant evolutionary relationships. As expected, the oldest Pfam domain age estimation strategy generates an older age distribution than the youngest Pfam domain strategy (Supplementary Figure S3). These patterns hold on average, but are not necessarily true for every protein. For all the analyses presented here, the differences between age estimation strategies shift the age distributions of proteins of interest without dramatically changing their relative orientation (e.g., Supplementary Figures S4 and S5); however, it is possible that other analyses could be more sensitive to these parameters.

### Implementation

*ProteinHistorian* consists of three python programs and a web server that provides an interface to many of their functions (Figure 1). The analyses provided on the web server can be extended and customized using the command line version of *ProteinHistorian*. The age_proteins.py program takes as input a species tree and a protein family database and uses an ancestral family reconstruction algorithm to compute protein ages as described above.

**Figure 3:**
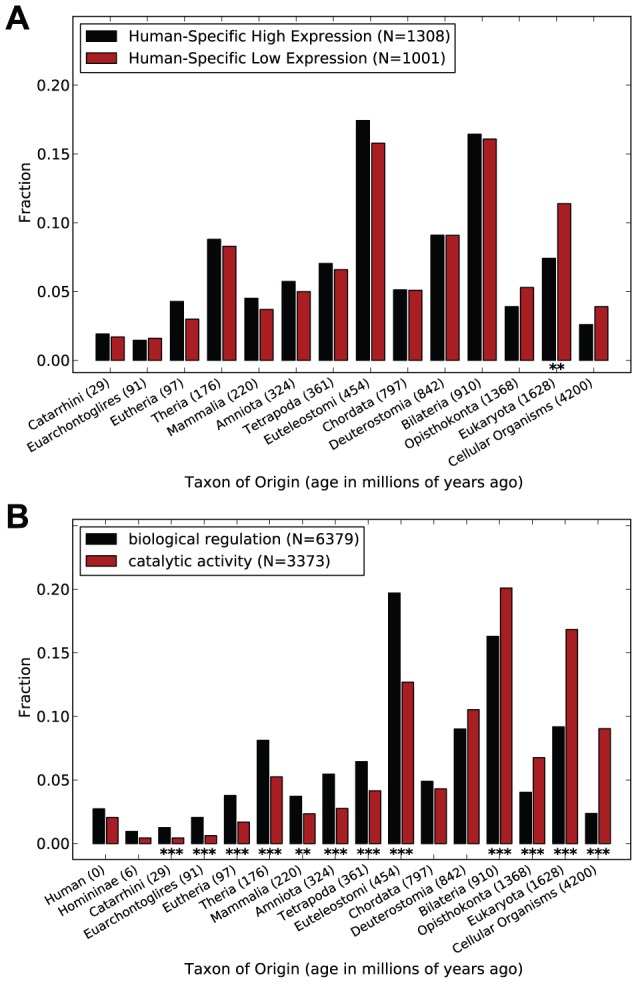
Protein age distribution comparisons. (**A**) Proteins with high expression on the human lineage (compared to non-human primates) have an average origin of 705.6 mya and are significantly younger than proteins with human-specific low expression (825.3 mya; Mann-Whitney U test: 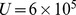; 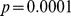). The most significant difference between the distributions is in the fraction of proteins created prior to the divergence of the Eukaryota (Fisher's exact test; *: 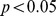; **: 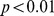; ***: 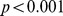). (**B**) Proteins with annotated regulatory functions are significantly younger (average age: 726.3 mya) than proteins with catalytic functions (average age: 1150.6 mya; Mann-Whitney U test: 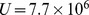; 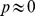).

**Figure 4:**
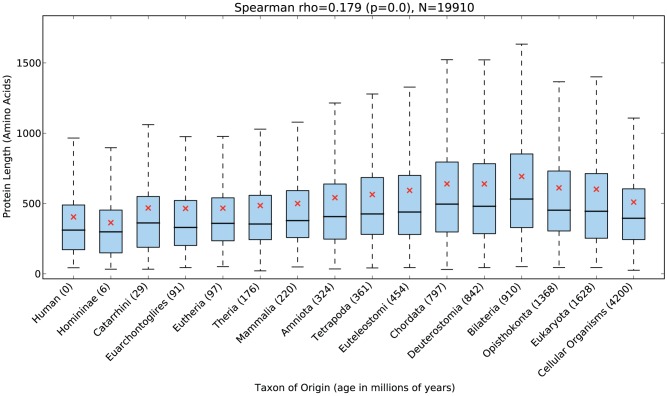
omparison of human protein age and length. The length of a human protein is significantly positively correlated with its age (Spearman 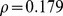; 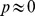). However, on average, the increase in age is not present in the most ancient age groups. Each blue box extends from the lower to the upper quartile of protein lengths observed for each age. The median age (bold horizontal black line), mean age (red x), and the minimum and maximum values observed within 1.5 times the interquartile range (whiskers) for each time point are also given. This pattern holds for a range of species (Table 1) and age estimation strategies (Supplementary Figure S5).

Given two sets of proteins of interest, age_enrichment_analysis.py uses the two-sided Mann-Whitney U test to test for a significant difference in the age distributions of the two sets. Significant differences in the fraction of proteins of a specific age from each set are detected using Fisher's exact test (Figure 3). When additional data about proteins are provided by the user, age_feature_analysis.py computes Spearman's rank correlation coefficient between the ages and features and draws box plots summarizing the distribution of the feature at each age (Figure 4).

The command line programs that perform all *ProteinHistorian* analyses are written in python v2.6.5. The freely available SciPy v0.7.0 [34], matplotlib v1.0.0 [35], and DendroPy v3.7.0 [36] python modules are required for full functionality. The Count [16] program is required for the use of asymmetric Wagner parsimony. More information about obtaining these dependencies, instructions for installing *ProteinHistorian*, and a tutorial on performing age analyses are given in the *ProteinHistorian* README.

The *ProteinHistorian* web server, which provides an interface to many of the functions performed by these programs, runs on an Ubuntu linux server running apache version 2.2. We used a combination of HTML, Perl, JavaScript, and R [37] to create the web interface, retrieve data from the user, send it to the command line programs, and produce the results pages.

The raw protein family data and age estimates for each species and database are all stored as tab-delimited text files. The species trees are stored in the Newick format. All data files are available for download on the web server.

## Results

The creation of *ProteinHistorian* was motivated by the potential for phylogenetic analysis to inform the study of protein function and disease. To illustrate the capabilities of *ProteinHistorian*, we now describe three example analyses.

### Proteins with human-specific high expression are significantly younger than proteins with human-specific low expression

A recent study used multi-species microarrays to quantify the expression levels of over 18,000 orthologous genes between human, chimpanzee, and rhesus macaque in liver, kidney, and heart tissue [38]. They used a linear mixed-effects model and a series of likelihood ratio tests to identify genes whose expression patterns showed signs of lineage-specific directional selection. These genes are of particular interest, because they are likely involved in producing the phenotypic differences that distinguish humans and our closest relatives.

We used *ProteinHistorian* to investigate whether there are differences in the phylogenetic origins of proteins found to have significantly higher expression patterns in human, compared to chimpanzee and rhesus macaque, than those under directional selection for lower expression. Comparing the overall age distributions for these two sets of proteins (Figure 3A), we find that proteins with unique high expression in human (in any of the tissues) are significantly younger than those with unique low expression (Mann-Whitney U test: 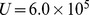; 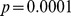). The average age of proteins with human-specific high expression is 705.6 mya, while the average for the human-specific low expression proteins is 825.3 mya. The difference between the distributions is largest in the oldest groups of proteins, with proteins created prior to the divergence of eukaryotes showing the most dramatic enrichment for proteins with human-specific low expression. This finding suggests that disrupting ancestral functions and expressing younger proteins may have contributed to the creation of human-specific traits. Further work is needed in order to understand the functional and evolutionary significance of the observed negative association between age and recent lineage-specific expression in humans.

The protein ages used in this analysis and all others in the text were estimated using Wagner parsimony on PPOD's OrthoMCL clustering of proteins in the PANTHER database. We observed similar patterns with other age estimation strategies and databases (Supplementary Figures S4 and S5). However, the particular age groups showing the most dramatic differences varied, as is expected given the different assumptions upon which the databases are built. We also note that 46 proteins included on the microarray as present in human, chimpanzee, and macaque were not present in all three species in the PPOD-PANTHER-OrthoMCL age database. We ignored these proteins in our analysis, but including them does not change the conclusions.

### Proteins with regulatory functions are significantly younger than those with catalytic functions

*ProteinHistorian* can be used to investigate the evolutionary history of pathways and to test hypotheses about the origin of proteins that share common functions. To facilitate these analyses, *ProteinHistorian* accepts protein lists in the Gene Ontology Annotation File (GAF) format 2.0 [21] as input—in addition to simple lists of protein names. For example, since differences in gene regulation are responsible for many of the phenotypic differences between species [39], we speculated that proteins with regulatory activities might be younger than proteins with other essential biochemical functions, such as catalysis. We tested this hypothesis by retrieving all human proteins with manual GO annotations to the “biological regulation” (GO:0065007) or “catalytic activity” (GO:0003824) categories using QuickGO [40] on January 7, 2012 and comparing their ages.

Regulatory proteins are significantly younger than catalytic proteins (Mann-Whitney U test: 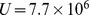; 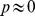; Figure 3B). Proteins in the catalytic activity category have an average age of 1150.6, while the regulatory proteins have an average age of 726.3 mya. Many catalytic proteins are ancient; over 25% are estimated to have been present in the last common ancestor of all eukaryotes. Significant differences between the fraction of proteins from the two sets are observed for the majority of the age groups. We observed similar patterns when we compared more fine-scale functional annotations within these categories, such as nucleic acid binding transcription factor activity and particular catalytic processes. To analyze more specific functional units, we used the Pfam domain age database to compare the age distribution of individual domains. We downloaded all domains that had “catalytic” or “regulat
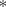
”, where 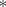 is a wildcard, in their description and compared their age distributions. The regulatory domains were also significantly younger than catalytic domains (Supplementary Figure S6; average age 1926.4 versus 3135.5 mya; Mann-Whitney U test: 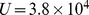; 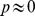).

### Old proteins are significantly longer than young proteins

In addition to performing protein age enrichment analysis, *ProteinHistorian* can correlate ages with other protein attributes. To illustrate this function, we compared protein age and length across the fungal and metazoan genomes present in the PPOD-PANTHER-OrthoMCL database. We limited our analysis to these species, because the other eukaryotic species did not have sufficient depth in the tree (Figure 2) to allow the estimation of high resolution ages.

Proteins with homologs across a diverse set of species have been anecdotally reported to be longer than proteins without evolutionarily distant homologs. This relationship has been observed in yeast, human, fly, and *Aspergillus* fungus [2], [6], but it has not been studied in depth. Our results confirm these previous observations and demonstrate the generality of this pattern. For example, the length of a human protein is significantly correlated (Spearman 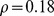; 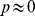) with its age (Figure 4).

A positive, though often relatively small in magnitude, correlation between protein age and length is present in all of the 24 species considered; the correlation is significant for 22 of the 24 species. (Table 1). The two exceptions are *Ashbya gossypii*, a filamentous fungus, which shows a very slight positive correlation (Spearman 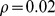; 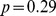) and *Schizosaccharomyces pombe*, fission yeast, which also shows a slight positive correlation (Spearman 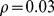; 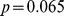). The strongest correlations are observed in mouse (0.32) and human (0.18). The magnitude of the correlation between age and length is often quite different between closely related species. For example, proteins in chimp (0.08) and rat (0.10) have lower correlations than human and mouse. We suspect that the comparatively strong correlation in human and mouse is due to these species having more extensively characterized proteomes than the other species considered. The predicted protein sets for most species are based, in part, on the existence of homologous proteins in other species, and so the less well annotated proteomes may be missing many lineage-specific proteins.

**Table 1:** Correlation of protein age and length across 24 fungi and metazoa.

Species	Spearman 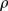	p-value	Number of Proteins
*Schizosaccharomyces pombe*	0.03	0.065	4987
*Aspergillus nidulans*	0.09	3.5e-17	9540
*Neurospora crassa*	0.12	1.5e-32	9820
*Ashbya gossypii*	0.02	0.29	4721
*Saccharomyces cerevisiae*	0.06	7.8e-06	5875
*Caenorhabditis briggsae*	0.06	4.7e-16	16330
*Caenorhabditis elegans*	0.14	4.5e-93	19986
*Anopheles gambiae*	0.12	9.5e-42	12456
*Drosophila melanogaster*	0.12	5.6e-48	13443
*Strongylocentrotus purpuratus*	0.14	3.1e-120	28605
*Ciona intestinalis*	0.15	4.6e-77	14179
*Danio rerio*	0.09	1e-40	21321
*Takifugu rubripes*	0.05	1.8e-10	18522
*Xenopus tropicalis*	0.07	2.7e-18	18022
*Gallus gallus*	0.07	3.9e-19	18228
*Ornithorhynchus anatinus*	0.10	1.3e-44	17950
*Monodelphis domestica*	0.06	4e-15	19470
*Canis familiaris*	0.10	6.1e-42	19304
*Bos taurus*	0.08	1.3e-34	21053
*Mus musculus*	0.32	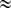 0	26184
*Rattus norvegicus*	0.10	3e-56	27757
*Macaca mulatta*	0.07	2.7e-22	21904
*Pan troglodytes*	0.08	2.1e-31	19828
*Homo sapiens*	0.18	1.8e-142	19910

Nearly all species show a significant, though often small, positive Spearman correlation between protein age and length. The two exceptions are *Schizosaccharomyces pombe* and *Ashbya gossypii*, for which the slight positive correlations are not significant. Human (Figure 4) and mouse show the strongest correlations overall.

Fitting a linear model to the human data suggests that proteins have increased in length by roughly 0.28 amino acids per million years on average since the origin of eukaryotes. We did not include the two most ancient groups—proteins with origins before the eukaryotes—in this analysis because they often did not maintain the increase in length. Several possibilities could explain the lack of a continuation of this pattern among the oldest proteins. Ancient proteins might be less subject the evolutionary processes driving increases in length, perhaps in part because many of them perform essential functions necessary for life. It is also possible that these older proteins have reached a natural limit on the length that proteins can maintain under normal circumstances.

## Availability and Future Directions

The *ProteinHistorian* tools enable biologists and bioinformaticians to perform powerful phylogenetic analyses on protein sets of interest across the eukaryotic tree of life. Using *ProteinHistorian* we found intriguing differences in the origins of proteins with increased versus decreased expression in humans; we tested a hypothesis about the age of regulatory proteins; and we demonstrated a very general positive correlation between protein age and length in fungi and metazoa.

The *ProteinHistorian* web server, source code, and data are freely available under the GNU public license. Because *ProteinHistorian* is easily extensible, we expect the tool to grow and develop as new data and algorithms become available. We hope to extend it to include analysis of prokaryotes by using ancestral reconstruction algorithms that can handle frequent horizontal gene transfer [16]. The *ProteinHistorian* framework could also be adapted to analyze the evolutionary origins of functional elements other than proteins.

## Supporting Information

Dataset S1 ***ProteinHistorian*** **source code and examples.** This archive contains the python source code for the command line version of *ProteinHistorian*. Installation instructions and several example analyses are given in the README file.
(ZIP)

Figure S1
**Dollo parsimony produces older protein age estimates than Wagner parsimony.** Each set of age estimates is based on a species tree, ancestral family reconstruction algorithm, and a protein family database. Different choices for each of these inputs will produce age distributions with different properties. For example, the Dollo parsimony ancestral reconstruction algorithm produces older ages for human proteins on average (average age: 1154.5 mya) than Wagner parsimony (average age: 681.4 mya; Mann-Whitney U test: 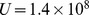; 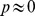). Dollo parsimony assumes that each protein family was only gained once, thus false positives in the family database and instances of horizontal gene transfer can inflate protein ages. In contrast, Wagner parsimony allows multiple gains in its reconstruction, and as a result, produces younger ages on average.(PDF)

Figure S2
**Jaccard Clustering produces older protein age estimates than OrthoMCL.** The PPOD protein family database based on Jaccard clustering produces older ages for human proteins on average (average age: 1289.1 mya) than the OrthoMCL-based database (average age: 817.9 mya; Mann-Whitney U test: 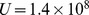; 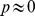). Jaccard clustering attempts to capture more distant evolutionary relationships than OrthoMCL, and this result suggests that it is successful. The family reconstruction for this analysis was performed with Dollo parsimony, but results are similar for Wagner parsimony (data not shown). Note that the species set used in this comparison is the 12 GO reference genomes, since there is not a Jaccard clustering family database available from PPOD for the full set of species in PANTHER.(PDF)

Figure S3
**Comparison of the age distributions resulting from the oldest Pfam domain and youngest Pfam domain age estimation strategies.** As expected, assigning human proteins the age of their oldest Pfam domain produces older ages on average (average age: 2433.7 mya) than assigning them the age of their youngest domain (average age: 1881.8 mya; Mann-Whitney U test: 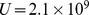; 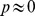).(PDF)

Figure S4
**Example comparisons of age distributions for protein sets of interest.** The comparisons of the age distributions of different protein sets of interest presented in the text (Figure 3) are similar when ancestral family reconstruction is performed using Dollo parsimony instead of Wagner parsimony. (**A**) Proteins with high expression on the human lineage (compared to non-human primates) have an average origin of 1215.4 mya and are significantly younger than proteins with human-specific low expression (1440.2 mya; Mann-Whitney U test: 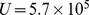; 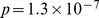). The distributions have significant differences in the fraction of proteins created around the divergence of Mammalia, Euteleostomi, Eukaryota, and all cellular life (Fisher's exact test; *: 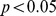; **: 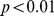; ***: 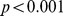). (**B**) Proteins with annotated regulatory functions are significantly younger (average age: 1150.7 mya) than proteins with catalytic functions (average age: 2025.7 mya; Mann-Whitney U test: 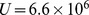; 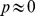).(PDF)

Figure S5
**Correlation between human protein age and length.** The length of a human protein is significantly positively correlated with its age (Spearman 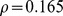; 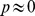) when using Dollo parsimony instead of Wagner parsimony. However, as in the Wagner analysis, the increase in age does not continue across the most ancient age groups. Each blue box extends from the lower to the upper quartile of protein lengths observed for each age. The median age (bold horizontal black line), mean age (red x), and the minimum and maximum values observed within 1.5 times the interquartile range (whiskers) for each time point are also given. This result holds across a range of species (Supplementary Table S1).(PDF)

Figure S6
**Comparison of Pfam catalytic and regulatory domain age distributions.** Pfam domains with catalytic activities are significantly older on average (average age: 3135.5 mya) than regulatory domains (average age: 1926.4 mya; Mann-Whitney U test: 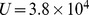; 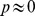). The domain groups were defined by searching for “catalytic” and “regulat” in the descriptions of all Pfam domains. Since all observed domains, not just those found in a single species, were considered in this analysis, the x-axis lists all possible taxa of origin.(PDF)

Table S1 **Correlation of protein age and length across 24 fungi and metazoa.** Using Dollo parsimony on the PPOD-PANTHER OrthoMCL database, the correlations between age and length are very similar to those reported in the main text (Table 1). Nearly all species show a significant, though often small, positive Spearman correlation between protein age and length. The one exception is *Ashbya gossypii*. Human (Figure 9) and mouse show the strongest correlations overall.
(PDF)
