# Supplementary figures and images for: ProteinHistorian: Tools for the Comparative Analysis of Eukaryote Protein Origin
Source: PLoS Comput Biol. 2012 Jun 28;8(6):e1002567. doi: 10.1371/journal.pcbi.1002567 (PMC3386163; doi:10.1371/journal.pcbi.1002567)

Mann-Whitney U test:  $U = 1.4e+08$  ( $p = 0$ )

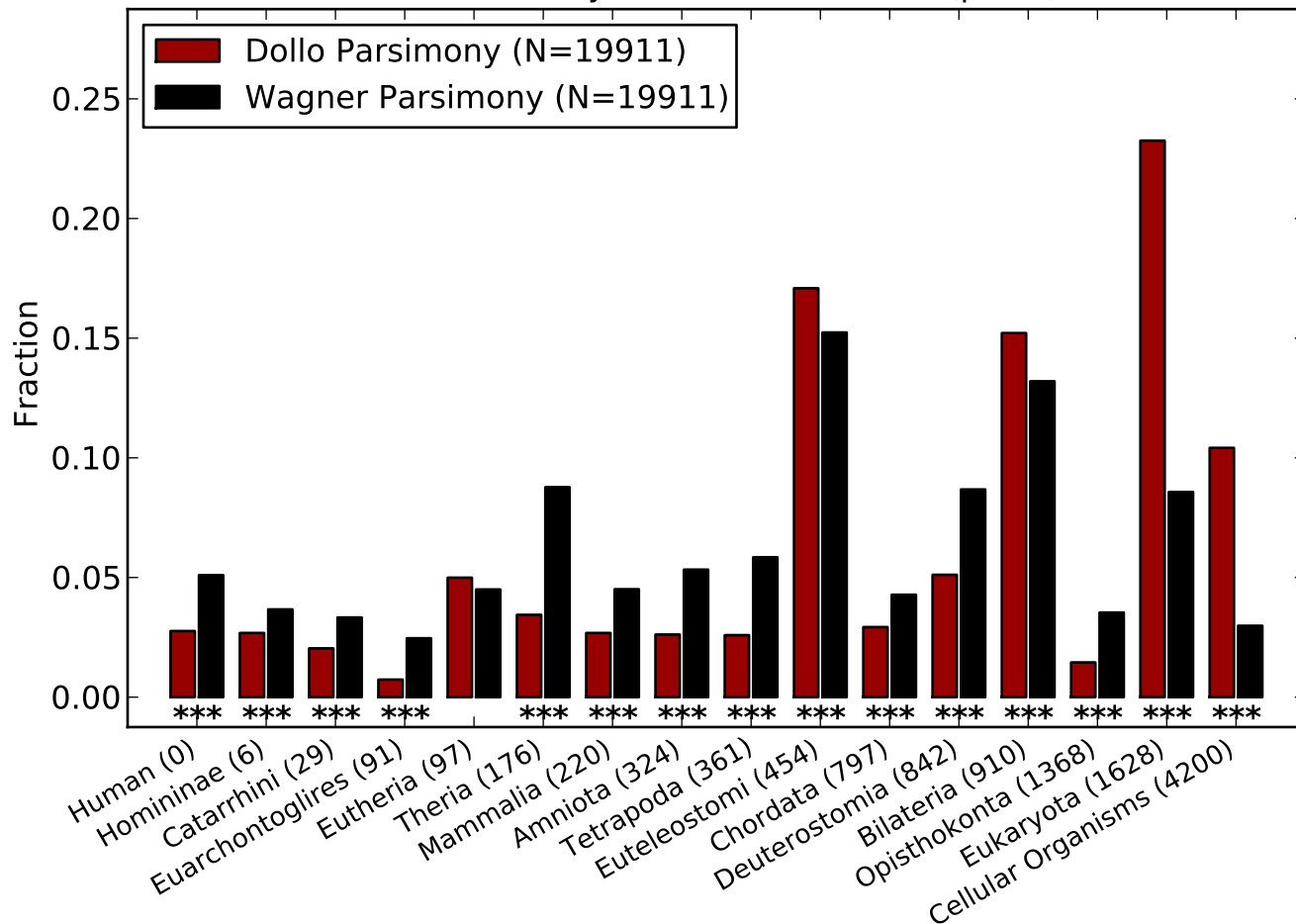

Taxon of Origin (age in millions of years ago)

Supplement: Figure S1 — Dollo parsimony produces older protein age estimates than Wagner parsimony. Each set of age estimates is based on a species tree, ancestral family reconstruction algorithm, and a protein family database. Different choices for each of these inputs will produce age distributions with different properties. For example, the Dollo parsimony ancestral reconstruction algorithm produces older ages for human proteins on average (average age: 1154.5 mya) than Wagner parsimony (average age: 681.4 mya; Mann-Whitney U test: ; ). Dollo parsimony assumes that each protein family was only gained once, thus false positives in the family database and instances of horizontal gene transfer can inflate protein ages. In contrast, Wagner parsimony allows multiple gains in its reconstruction, and as a result, produces younger ages on average. (PDF) [file pcbi.1002567.s002.pdf]

Mann-Whitney U test:  $U = 1.4e+08$  ( $p = 0$ )

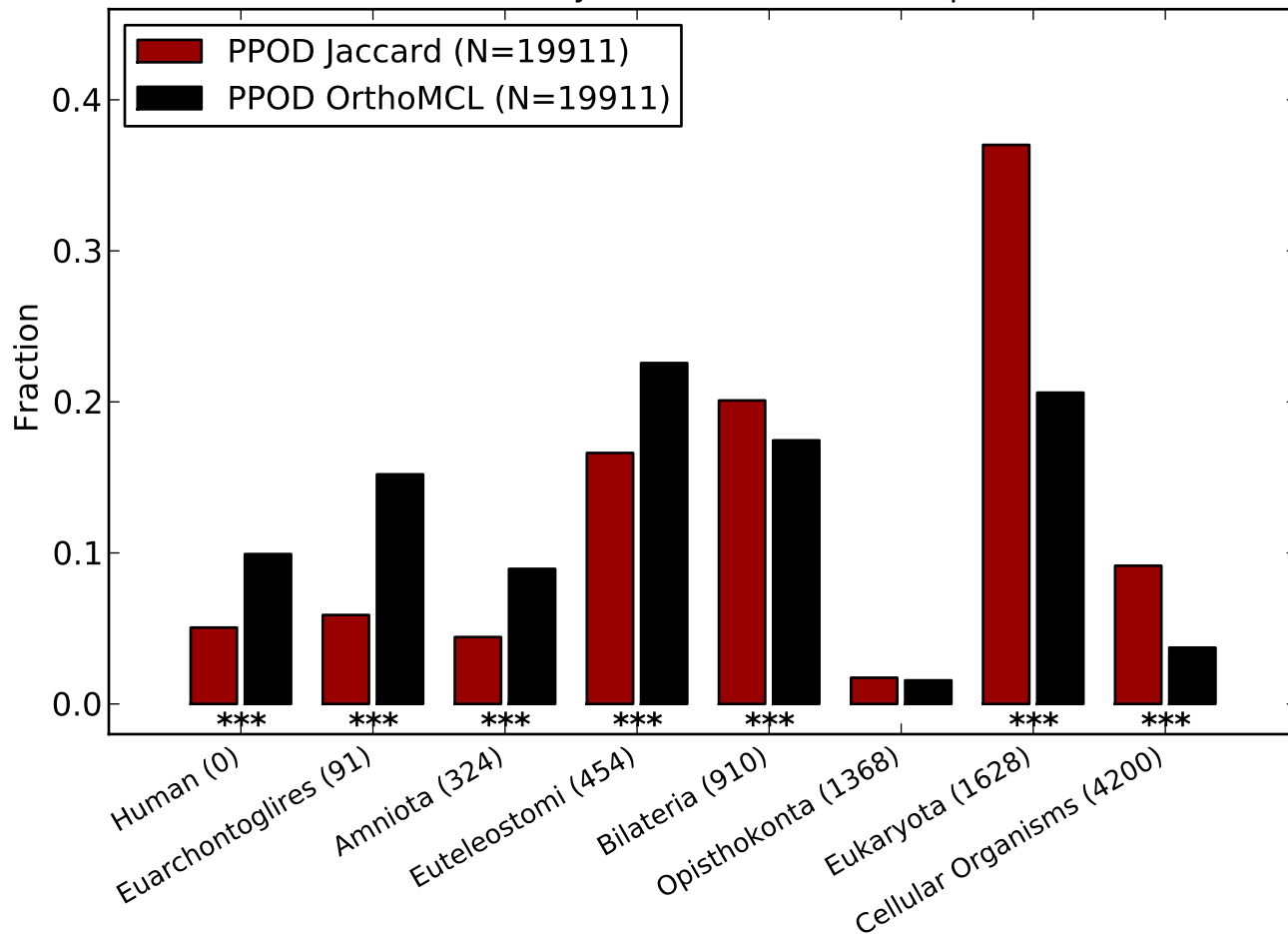

Taxon of Origin (age in millions of years ago)

Supplement: Figure S2 — Jaccard Clustering produces older protein age estimates than OrthoMCL. The PPOD protein family database based on Jaccard clustering produces older ages for human proteins on average (average age: 1289.1 mya) than the OrthoMCL-based database (average age: 817.9 mya; Mann-Whitney U test: ; ). Jaccard clustering attempts to capture more distant evolutionary relationships than OrthoMCL, and this result suggests that it is successful. The family reconstruction for this analysis was performed with Dollo parsimony, but results are similar for Wagner parsimony (data not shown). Note that the species set used in this comparison is the 12 GO reference genomes, since there is not a Jaccard clustering family database available from PPOD for the full set of species in PANTHER. (PDF) [file pcbi.1002567.s003.pdf]

Mann-Whitney U test:  $U = 2.1e+09$  ( $p = 0$ )

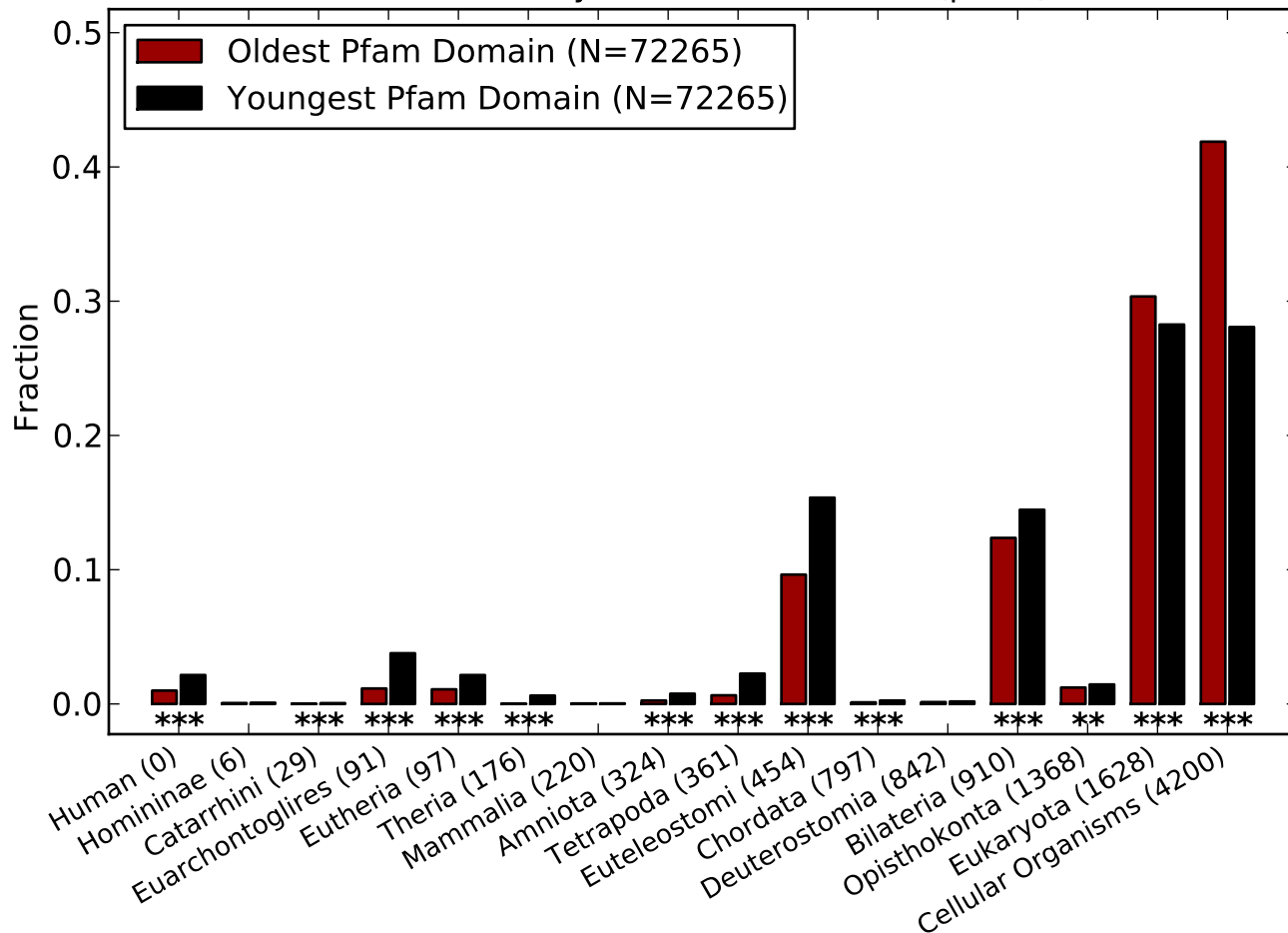

Taxon of Origin (age in millions of years ago)

Supplement: Figure S3 — Comparison of the age distributions resulting from the oldest Pfam domain and youngest Pfam domain age estimation strategies. As expected, assigning human proteins the age of their oldest Pfam domain produces older ages on average (average age: 2433.7 mya) than assigning them the age of their youngest domain (average age: 1881.8 mya; Mann-Whitney U test: ; ). (PDF) [file pcbi.1002567.s004.pdf]

**A**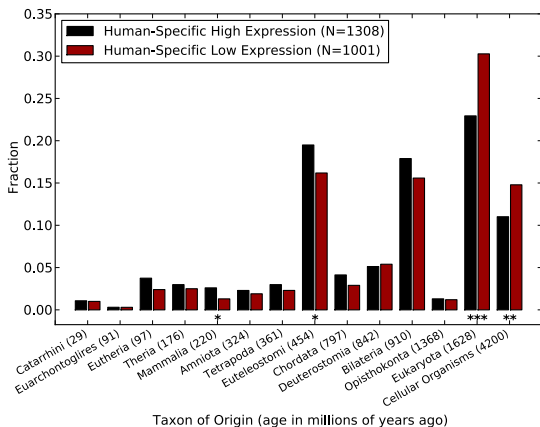**B**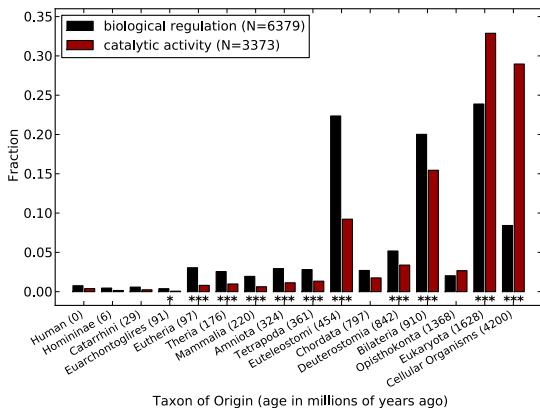

Supplement: Figure S4 — Example comparisons of age distributions for protein sets of interest. The comparisons of the age distributions of different protein sets of interest presented in the text (Figure 3) are similar when ancestral family reconstruction is performed using Dollo parsimony instead of Wagner parsimony. (A) Proteins with high expression on the human lineage (compared to non-human primates) have an average origin of 1215.4 mya and are significantly younger than proteins with human-specific low expression (1440.2 mya; Mann-Whitney U test: ; ). The distributions have significant differences in the fraction of proteins created around the divergence of Mammalia, Euteleostomi, Eukaryota, and all cellular life (Fisher's exact test; *: ; **: ; ***: ). (B) Proteins with annotated regulatory functions are significantly younger (average age: 1150.7 mya) than proteins with catalytic functions (average age: 2025.7 mya; Mann-Whitney U test: ; ). (PDF) [file pcbi.1002567.s005.pdf]

Spearman rho=0.165 (p=0.0), N=19910

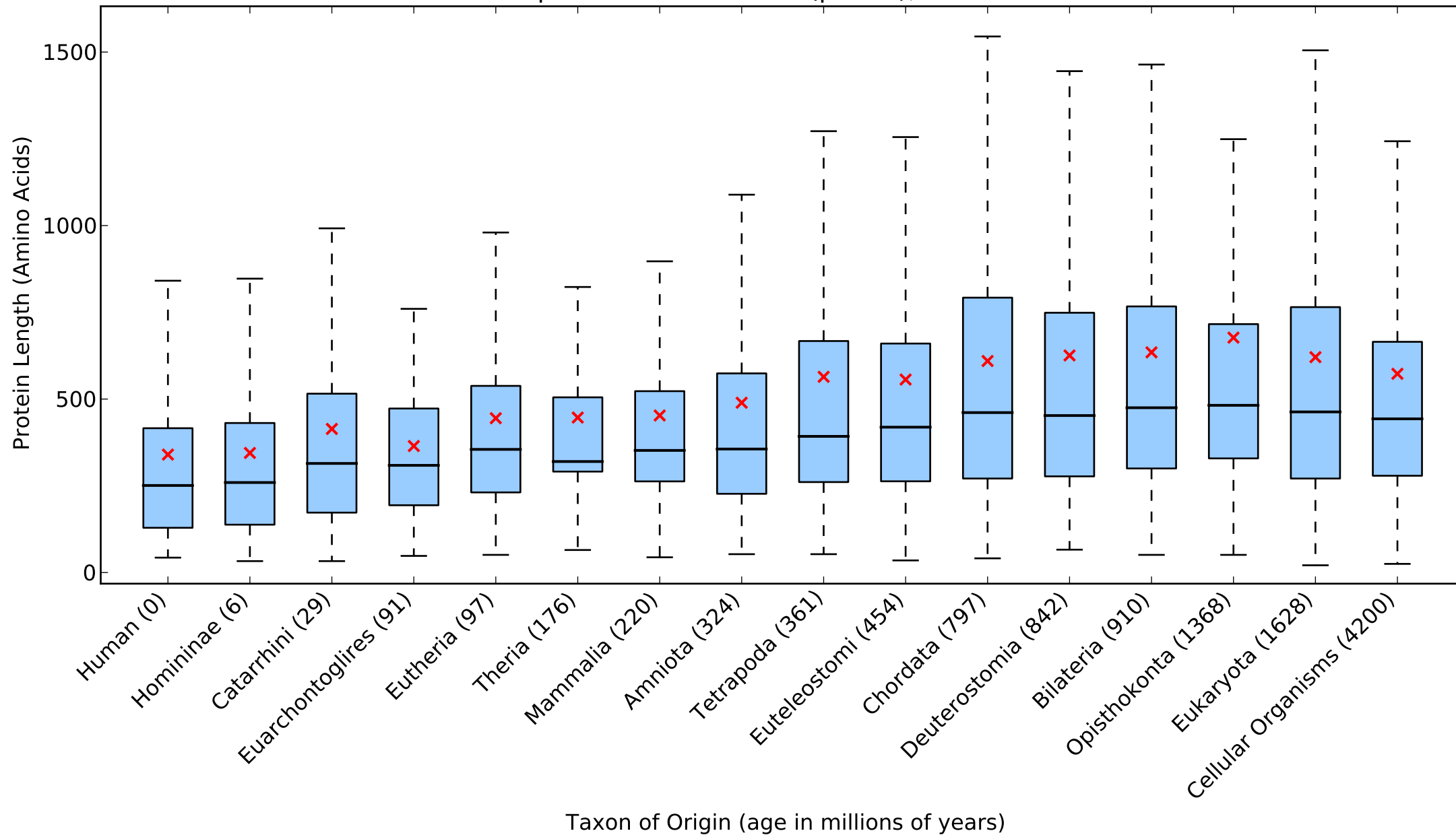

Supplement: Figure S5 — Correlation between human protein age and length. The length of a human protein is significantly positively correlated with its age (Spearman ; ) when using Dollo parsimony instead of Wagner parsimony. However, as in the Wagner analysis, the increase in age does not continue across the most ancient age groups. Each blue box extends from the lower to the upper quartile of protein lengths observed for each age. The median age (bold horizontal black line), mean age (red x), and the minimum and maximum values observed within 1.5 times the interquartile range (whiskers) for each time point are also given. This result holds across a range of species (Supplementary Table S1). (PDF) [file pcbi.1002567.s006.pdf]

Mann-Whitney U test:  $U = 3.8e+04$  ( $p = 0$ )

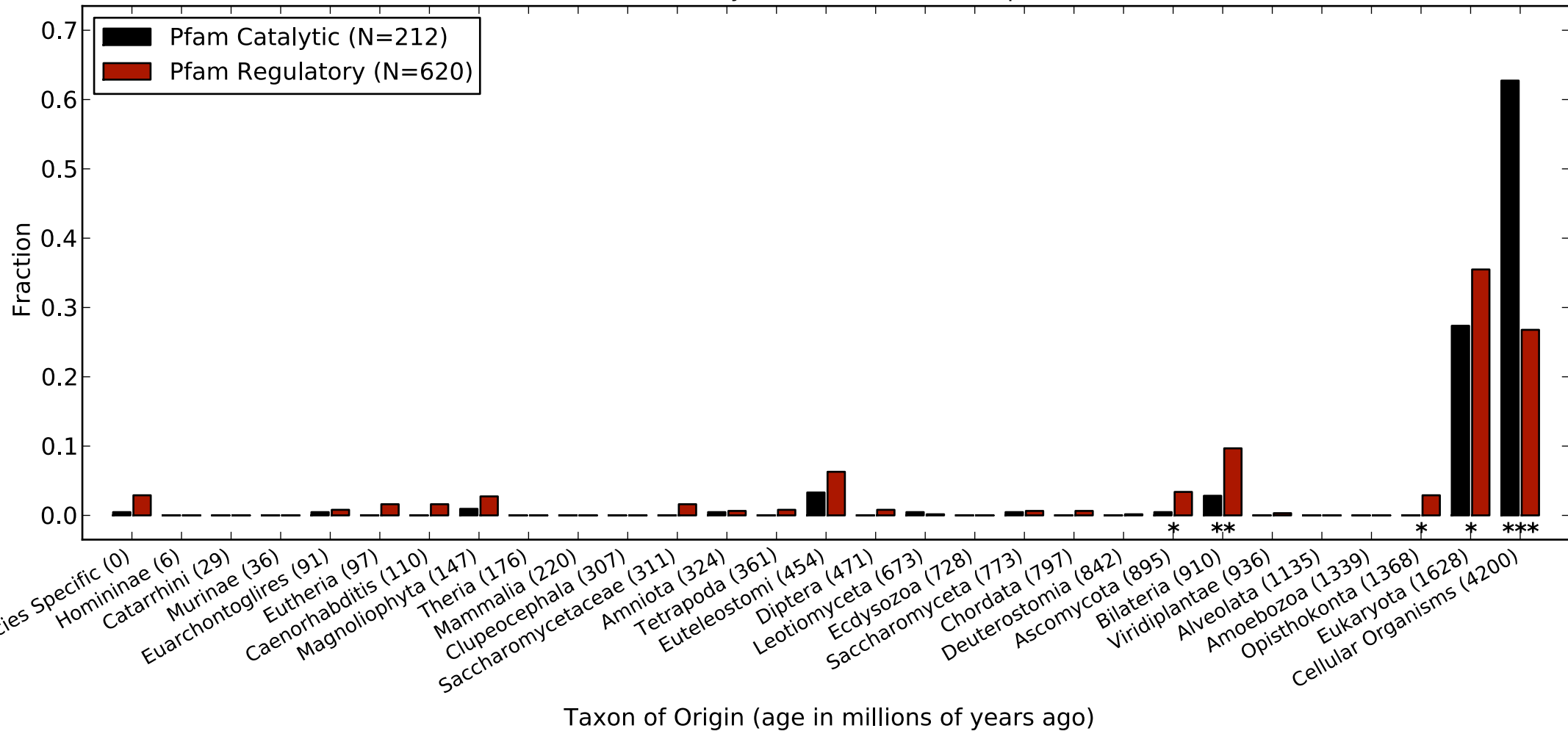

Supplement: Figure S6 — Comparison of Pfam catalytic and regulatory domain age distributions. Pfam domains with catalytic activities are significantly older on average (average age: 3135.5 mya) than regulatory domains (average age: 1926.4 mya; Mann-Whitney U test: ; ). The domain groups were defined by searching for “catalytic” and “regulat” in the descriptions of all Pfam domains. Since all observed domains, not just those found in a single species, were considered in this analysis, the x-axis lists all possible taxa of origin. (PDF) [file pcbi.1002567.s007.pdf]
