# Supplementary material for: ProteinHistorian: Tools for the Comparative Analysis of Eukaryote Protein Origin
Source: PLoS Comput Biol. 2012 Jun 28;8(6):e1002567. doi: 10.1371/journal.pcbi.1002567 (PMC3386163; doi:10.1371/journal.pcbi.1002567)
Supplement: Table S1 — Correlation of protein age and length across 24 fungi and metazoa. Using Dollo parsimony on the PPOD-PANTHER OrthoMCL database, the correlations between age and length are very similar to those reported in the main text (Table 1). Nearly all species show a significant, though often small, positive Spearman correlation between protein age and length. The one exception is Ashbya gossypii. Human (Figure 9) and mouse show the strongest correlations overall. (PDF) [file pcbi.1002567.s008.pdf]

Table S1: **Correlation of protein age and length across 24 fungi and metazoa**

Species	Spearman ρ	p-value	Number of Proteins
Schizosaccharomyces pombe	0.05	0.0013	4987
Aspergillus nidulans	0.06	1.7e-09	9540
Neurospora crassa	0.11	1.1e-29	9820
Ashbya gossypii	0.03	0.055	4721
Saccharomyces cerevisiae	0.07	1.9e-08	5875
Caenorhabditis briggsae	0.04	9e-08	16330
Caenorhabditis elegans	0.13	1.8e-78	19986
Anopheles gambiae	0.12	1.1e-38	12456
Drosophila melanogaster	0.12	4.6e-42	13443
Strongylocentrotus purpuratus	0.06	1.2e-20	28605
Ciona intestinalis	0.12	4e-50	14179
Danio rerio	0.09	8.9e-36	21321
Takifugu rubripes	0.06	1e-13	18522
Xenopus tropicalis	0.04	7.7e-08	18022
Gallus gallus	0.06	1.8e-17	18228
Ornithorhynchus anatinus	0.11	2.2e-48	17950
Monodelphis domestica	0.07	3.8e-23	19470
Canis familiaris	0.10	1.7e-47	19304
Bos taurus	0.09	3.7e-36	21053
Mus musculus	0.31	$\approx$0	26184
Rattus norvegicus	0.09	2.9e-47	27757
Macaca mulatta	0.07	3.5e-23	21904
Pan troglodytes	0.09	4.8e-36	19828
Homo sapiens	0.17	4.4e-122	19910

Using Dollo parsimony on the PPOD-PANTHER OrthoMCL database, the correlations between age and length are very similar to those reported in the main text. Nearly all species show a significant, though often small, positive Spearman correlation between protein age and length. The one exception is *Ashbya gossypii*. Human (Figure S5) and mouse show the strongest correlations overall.
